# Atomic Resolution Insights into Curli Fiber Biogenesis

**DOI:** 10.1016/j.str.2011.05.015

**Published:** 2011-09-07

**Authors:** Jonathan D. Taylor, Yizhou Zhou, Paula S. Salgado, Ardan Patwardhan, Matt McGuffie, Tillmann Pape, Grzegorz Grabe, Elisabeth Ashman, Sean C. Constable, Peter J. Simpson, Wei-chao Lee, Ernesto Cota, Matthew R. Chapman, Steve J. Matthews

**Affiliations:** 1 Division of Molecular Biosciences, Faculty of Natural Sciences, Imperial College London, London, SW7 2AZ, UK; 2 Department of Molecular, Cellular, and Developmental Biology, University of Michigan, Ann Arbor, MI 48109, USA

## Abstract

Bacteria produce functional amyloid fibers called curli in a controlled, noncytotoxic manner. These extracellular fimbriae enable biofilm formation and promote pathogenicity. Understanding curli biogenesis is important for appreciating microbial lifestyles and will offer clues as to how disease-associated human amyloid formation might be ameliorated. Proteins encoded by the curli specific genes (*csgA-G*) are required for curli production. We have determined the structure of CsgC and derived the first structural model of the outer-membrane subunit translocator CsgG. Unexpectedly, CsgC is related to the N-terminal domain of DsbD, both in structure and oxido-reductase capability. Furthermore, we show that CsgG belongs to the nascent class of helical outer-membrane macromolecular exporters. A cysteine in a CsgG transmembrane helix is a potential target of CsgC, and mutation of this residue influences curli assembly. Our study provides the first high-resolution structural insights into curli biogenesis.

## Graphical abstract

**Graphical Abstract:**

**Figure:**
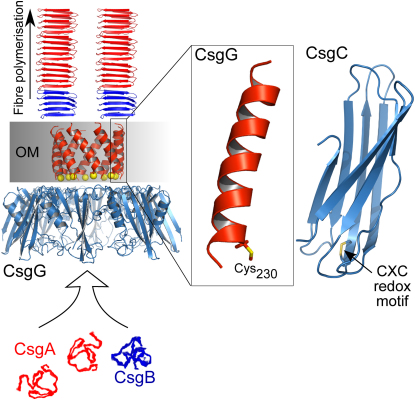

**Highlights:**

► CsgC is related to the redox-active N-terminal domain of DsbD ► Outer-membrane transporter CsgG inserts into the membrane via an α-helical oligomer ► The transmembrane domain of CsgG is vital for pore forming and curli assembly ► CsgC appears to affect CsgG pore behavior and biofilm formation

## Introduction

A wide variety of bacteria are now known to create protective fibers at their surface utilizing amyloid structure, as exemplified by the curli system (Barnhart and Chapman, 2006; Larsen et al., 2007). In enteric bacteria such as *Escherichia*, these fibers are 5–12 nm in diameter and varying lengths in the micrometre range (Olsén et al., 1989). Bacteria decorated by curli fibers tend to be auto-aggregative, form red-dry-and-rough (rdar) colonies on Congo red plates, and readily adopt biofilm lifestyles (Austin et al., 1998; Collinson et al., 1991). As well as this protective effect, curli fibers can adhere to a variety of circulating and immobilized host proteins or abiotic surfaces (Boyer et al., 2007; Gophna et al., 2002; Pawar et al., 2005). Host interactions induce the release of proinflammatory cytokines and activate nitric oxide synthase (NOS) 2 expression, causing inflammation and hypotension in mice (Bian et al., 2001; Tükel et al., 2009).

Curli fibers are almost entirely comprised of the curli specific gene subunit protein CsgA and are highly resistant to chemical or proteolytic degradation (Collinson et al., 1991). The five imperfect sequence repeats of CsgA are predicted to fold into a compact β-helix capable of self-oligomerization into an amyloid fiber (Chapman et al., 2002; Collinson et al., 1999; Shewmaker et al., 2009). In most γ-proteobacteria, genes encoding up to five accessory proteins enable specific secretion of unfolded CsgA and nucleation into a fiber. The main player in this potentially cytotoxic process is CsgG, an oligomeric, outer-membrane translocator through which CsgA is secreted. Until now, we have had little understanding of the structure or secretion mechanism of CsgG. Associated with this pore are two smaller proteins, CsgE and CsgF, which are necessary for subunit stability and cell-association of the fibers, respectively (Nenninger et al., 2009; Robinson et al., 2006). A second CsgA-like protein, known as CsgB, is required for nucleating or templating CsgA polymerization into an amyloid fiber (Hammer et al., 2007; White et al., 2001). CsgB is also translocated by CsgG and, probably through association with CsgF, adopts an amyloid-like conformation that templates CsgA aggregation (Hammer et al., 2007; Nenninger et al., 2009).

The role of the final component of the curli system, CsgC, remains unexplained. Although a rapidly growing number of bacterial species are now known to produce amyloid fibers, the CsgC accessory protein is found exclusively within a group of closely-related *Enterobacteriaceae* including *Escherichia* and *Salmonella*. Mutant strains lacking *csgC* still assemble curli yet they show defects in auto-aggregation, form paler colonies on Congo red plates, and display variable affinity for soluble fibronectin (Hammar et al., 1995). Studies of the CsgC homolog from *Salmonella,* AgfC, resulted in several intriguing observations (Gibson et al., 2007). First, loss of *agfC* caused an increase in fiber diameter, with an abundance of extremely durable 20 nm fibers alongside the usual 5–7 nm fibers. Although the AgfA monomers reacted with AgfA-specific polyclonal antisera, they were not strongly recognized using an AgfA mAb that recognizes a conformational epitope. This suggests that within the *agfC* mutant the AgfA subunits had adopted a different tertiary structure. Second, unlike wild-type fibers in which the ratio of AgfA to AgfB is ∼20:1 (White et al., 2001), the aberrant fibers completely lacked AgfB implying that it does not nucleate the alternative form of AgfA. Further experiments showed that in the absence of *agfC*, secreted AgfA subunits cannot be captured by anti-AgfA serum (Gibson et al., 2007). This supports the notion that AgfC affects the structure of AgfA during biogenesis.

In this study, we report the crystal structure of CsgC, the first among the Csg proteins to be solved. The protein adopts an immunoglobulin-like fold and contains an unusual invariant CxC motif reminiscent of the oxido-reductase superfamily CxxC motif. Based on its similarity to the N-terminal domain of DsbD, we propose that CsgC has redox activity and CsgG is the potential substrate. We also analyzed the structure of CsgG and its oligomerization and our results suggest the predicted transmembrane domain plays a role in curli assembly.

## Results and Discussion

### CsgC Is Related to DsbD

**Figure 1:**
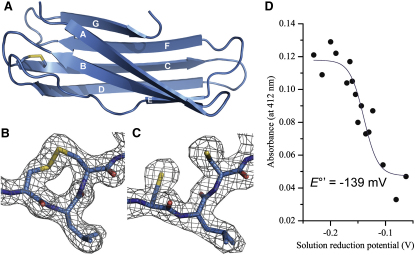
Atomic Structure of CsgC (A) Cartoon representation of the structure of oxidized CsgC. Strands are labeled according to standard Ig-fold nomenclature. The disulfide bond connecting C29 to C31 is shown as yellow sticks. (B and C) Electron density maps contoured at 1.5× σ level of CsgC in the oxidized and reduced states, respectively. (D) Reduction potential of CsgC. The absorbance at 412 nm, which correlates with the amount of free thiols reactive to DTNB, is plotted against the solution reduction potential. The data were fitted to the Nernst equation to yield a reduction potential of −139 ± 6 mV. See also Figures S1 and S3.

Scientific interest in curli has focused on the discovery and characterization of “functional amyloid” (Chapman et al., 2002; Wang and Chapman, 2008). To date there have been comparatively few studies of the proteins that guide fiber formation; for example, the role of the periplasmic protein CsgC during fiber biogenesis is unknown. To help define a role for CsgC we solved the X-ray crystal structure of the *Escherichia coli* form to 2.4 Å (Table 1; see Table S1 available online). The overall structure of CsgC consists of an immunoglobulin (Ig)-like β sandwich with seven strands forming two sheets and well-defined loop regions (Figure 1A). The extreme C-terminal region of CsgC projects away from the protein surface leading to a lack of electron density for the final six residues. Nuclear magnetic resonance (NMR) relaxation measurements confirmed the increased flexibility of this region (Figure S1).

**Figure 2:**
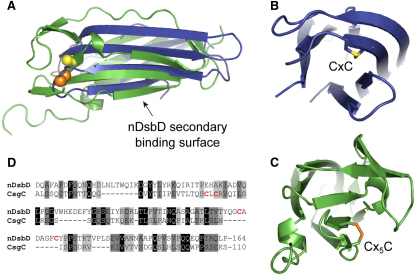
Comparison of the Structures of Oxidized CsgC and the N-Terminal Domain of DsbD (A) Structural alignment of CsgC (blue) and nDsbD (green). The RMS difference of Cα positions in the core Ig fold is 4.2 Å. Cysteine sulfur atoms are shown as spheres and colored yellow (CsgC) or orange (nDsbD). The distance between the disulfides within the structural alignment is ∼6 Å. (B and C) show aligned end views of CsgC and nDsbD, respectively, highlighting structural differences that will affect target specificity and reduction potential. (D) Sequence alignment of the core Ig folds of CsgC and nDsbD indicate 16% identity and 36% similarity. The shading of the residue background is in proportion to the chemical similarity of the side chains (i.e., black = identical, white = very different). The key cysteines are highlighted in red. The alignment was performed by ClustalW2 (www.ebi.ac.uk/Tools/msa/clustalw2) according to default parameters. See also Figures S2 and S4.

A unique feature of the CsgC structure is the invariant CxC motif that connects the BC loop to strand C (Figure 1A). The cysteine residues are located in the “complementarity-determining region” (CDR) of the Ig fold, a common site of target recognition, suggesting that they could be key to the cellular function of CsgC. A search for redox-active Ig folds highlighted the only other known example—the *E. coli* inner-membrane protein DsbD (Goulding et al., 2002). This protein is a redox hub that reduces periplasmic oxido-reductases such as DsbC, DsbG, and CcmG (Ito and Inaba, 2008). Cytoplasmic thioredoxin donates electrons to the integral membrane domain of DsbD, which transfers them to its periplasmic N-terminal, Ig-like domain (nDsbD) via its C-terminal, thioredoxin-like domain. Structural alignment of nDsbD (PDB code 1L6P) and CsgC reveals a close match in overall structure and, crucially, the location of the key cysteine pairs of each protein (Figure 2). The nDsbD domain contains a Cx_5_C motif within the FG loop, and the cysteines are capped by a phenylalanine that controls substrate specificity. Similarly, the CxC motif of CsgC is partially buried and the intervening side chain is always an exposed, nonpolar residue (L/V). It is interesting to note that although the amino acid sequence identity shared between *E. coli* nDsbD and CsgC is relatively low, this masks a much higher conservation at the genetic level suggesting evolution from a common ancestor (Figure S2). Moreover, from a protein folding point of view it is remarkable that despite the presence of multiple reading-frame shifts due to nucleotide insertions/deletions, the expressed proteins adopt the same overall structure and redox function.

Interestingly, nDsbD is known to recognize substrates using two binding sites—the cysteine loop and the flat, upper surface of the Ig fold formed by the N- and C-terminal strands of the domain. Although it is not known if CsgC also makes use of this secondary interaction site we found that the opposite, lower surface formed by strands D and E was the site of an dimer interface with the crystal lattice and displayed structural flexibility that prevented detection of several backbone amide resonances within NMR spectra (Figure S3).

Finally, from S_N_2 theory of thiol-disulfide exchange reactions, it is expected that the transition state involves linear alignment of the three participating sulfur atoms (Wiita et al., 2007). In this regard, we note that the disulfide bond within CsgC points directly out of a shallow pocket enabling these optimal interactions to occur with its target.

### Biochemical Analysis of the CxC Motif

Due to the overall similarity of CsgC to nDsbD we hypothesize that CsgC is involved in redox activity within the curli biogenesis system. Assignment of an enzymatic activity is also consistent with previous reports in which extremely low levels of CsgC were detected (Gibson et al., 2007). We obtained structures of CsgC in which the cysteines were either oxidized or reduced (Figures 1B and 1C). There were no gross structural changes associated with disulfide bond formation (root-mean-square deviation [rmsd] Cα backbone atoms = 0.65 Å), which is consistent with the high degree of similarity observed between NMR spectra of reduced and oxidized CsgC (Figure S4). In order to confirm the oxidation state of CsgC in vitro, mass spectrometry was used to measure accurate molecular weights for the oxidized and reduced forms. The reduced protein had a molecular weight of 12,066 Da, however after incubation with oxidized glutathione (GSSG) this decreased by 2 Da, indicating formation of the disulfide (Figure S4C). Strikingly, the introduction of the disulfide bond caused a large increase in protein stability, as evidenced by a shift in the thermal melting midpoint *T*_m_ value from 66.1 ± 0.5°C to 92.4 ± 0.8°C (Figure S4D).

We also measured the reduction potential (*E*°′) of CsgC to a value of −139 ± 6 mV (Figure 1D), which is more oxidizing than that measured for nDsbD (−229 mV) (Collet et al., 2002). Instead, CsgC is closer to the protein disulfide isomerases (PDI) DsbC (−126 mV) and DsbG (−130 mV) (Bessette et al., 1999; Zapun et al., 1995). Reports of other CxC motifs indicate similar reduction potentials; for instance, the tripeptide CGC (−167 mV), a thioredoxin CGC mutant (−200 mV) and bacterial PDI YphP (−130 mV) (Derewenda et al., 2009; Woycechowsky and Raines, 2003). It is therefore highly likely that CsgC reacts with specific thiols or disulfides.

### C230 of CsgG Is a Putative Target for CsgC

The intriguing presence of the CxC motif in CsgC and its potential reactivity toward cysteines prompted us to search for substrates within the other curli proteins. The only protein that contains conserved cysteines is the subunit translocator CsgG, which has two, C16 and C230. The former becomes the lipidated, N terminus of the mature protein (Loferer et al., 1997). It is unlikely that the function of CsgC is to attack the thioester bond between C16 and its cognate lipid because this posttranslational modification is essential for outer-membrane attachment of CsgG (Robinson et al., 2006). The latter site (C230) is conserved within most *Enterobacteriaceae* and a few other γ-proteobacteria. Indeed phylogenetic analysis shows C230 to be present in all species that have a *csgC* gene, but is nearly always mutated to a small amino acid (A, S, or G) in species lacking *csgC*. Cysteine is underrepresented in proteins that function in oxidizing environments and a strong bias exists toward even numbers within the same protein (Dutton et al., 2008). Therefore, the single conserved cysteine in CsgG suggests it may make a beneficial contribution to structure or function via changes in redox status that could be influenced by CsgC.

### Prediction of CsgG Transmembrane Region

The biological importance of CsgG as a secretion system is underlined by its conservation across the majority of known species within the bacterial kingdom. Knowledge of its atomic structure would enable much clearer understanding of the mechanisms by which it recognizes its protein substrates and performs translocation in the apparent absence of an energy source, such as ATP or a chemical gradient. We have used a combination of bioinformatics and in vitro studies to derive a homology model of CsgG that allows us to make predictions, with particular focus on the residue of interest, C230.

CsgG is an oligomeric, outer-membrane lipoprotein that facilitates export of curli fiber subunits and acts as a scaffold for nucleation (Epstein et al., 2009; Loferer et al., 1997; Robinson et al., 2006). Amino acid sequence analysis indicates that CsgG is highly unlikely to adopt the β-barrel fold that is almost always observed in bacterial outer-membrane protein structures. Indeed we find that recombinant CsgG_16-277_ can be purified from the soluble fraction of *E. coli* in a folded, but polydispersed oligomeric state in the absence of detergents (Figure S5A). Recently a second class of outer-membrane proteins has been discovered that utilizes α helices to span the membrane instead of β strands (Beis et al., 2004; Chandran et al., 2009; Kowalska et al., 2010; Meng et al., 2009). To date, the proteins within this class (Wza, TraF, PelC) all form oligomers and facilitate export of macromolecules. On the basis of our current knowledge we predict that CsgG is a member of this nascent group of outer-membrane proteins.

Using a combination of bioinformatic analyses we identified a 20-residue region (226–245) close to the C terminus of CsgG that is likely to form a transmembrane (TM) helix (see Figure S6). This region shows a conserved hydrophobic character and includes motifs that are enriched in helices capable of self-interaction, such as [GA]-X_3_-[GA] and [IV]-X_3_-[IVL], where X is any side chain (Senes et al., 2000). Additional copies of these motifs occur in residues 245–254, thus potentially extending the helix by a further three turns above the outer membrane surface (Figure S6I).

CsgG has been shown to form homo-oligomeric pores of undetermined stoichiometry within the outer-membrane (Robinson et al., 2006). We used size-exclusion chromatography and blue native PAGE to estimate for the first time the stoichiometry of the complex (Figures S5B and S5C). Taking into account hydrodynamic effects from the bound detergent, our data indicate that the number of CsgG protomers within the oligomer is between seven and nine. Recombinant CsgG-6xHis displays some polydispersity caused by self-association of oligomers, observable in size-exclusion and native PAGE (Figure S5). Given the potential for error in measuring stoichiometry in protein-detergent complexes using these methods, we also performed analytical gel filtration with nonmembrane-targeted CsgG_16-277_ (Figure S5A). The results were similar to that observed for the protein-detergent complex and support an assignment of octameric stoichiometry for CsgG.

**Figure 3:**
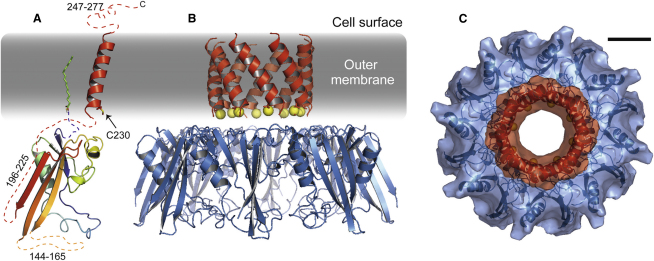
Homology Model of CsgG (A) Predicted structure of a CsgG monomer based on the N-terminal domain of TolB (PDB code 2HQS). The periplasmic domain (rainbow colors, N to C terminus) is attached to the outer membrane by both N-terminal palmitoylation (shown as sticks) and the C-terminal transmembrane helix. The short regions of CsgG that were omitted from the model by PHYRE are shown as dashed lines, with missing residue numbers shown in italics. The location of C230 is indicated. (B) Model of the CsgG oligomer, with periplasmic domain monomers (blue) arranged as observed in the structurally-related protein DUF330 (PDB code 2IQI). The transmembrane domain (red) was modeled on Wza (PDB code 2J58). The sulfur atoms of C230 are depicted as yellow spheres. (C) View of the CsgG oligomer observed from the outside of the cell showing a pore of ∼20 Å diameter. The solvent-accessible molecular surface was calculated by PyMOL (Delano Scientific). Scale bar represents 20 Ǻ. See also Figures S5 to S7.

The related protein Wza also consists of an octameric helical pore thus we created a homology model of the transmembrane region of CsgG using the Wza coordinates as a template (Figure 3). A helical wheel projection indicates that the TM region of CsgG is amphipathic (Figure S6H), which through oligomerization would expose nonpolar side chains to the hydrophobic lipid bilayer and create a hydrophilic surface within the pore. In this arrangement, the conserved cysteine residue (C230) would project into the lumen of the pore at the periplasmic entrance.

To test if the predicted TM region, especially the C230 residue, is important to CsgG stability and function we constructed a series of mutated forms of CsgG. First, we found that expression of a truncated form of CsgG lacking its TM helix and short C-terminal region (i.e., CsgG_Δ227-277_) was unable to rescue curli formation (as evidenced by lack of Congo red staining) in a *csgG* mutant strain (data not shown). A similar loss of function was observed when the predicted C-terminal amphipathic helix of PelC was deleted (Kowalska et al., 2010). Next we mutated two valine residues that we hypothesize are involved in helix-helix interactions. A V227A mutant was expressed at lower levels than wild-type and retained surface exposure however no Congo red staining was observed indicating that this residue is important to the proper function of CsgG. A V239A mutant was strongly destabilizing, and again apparently produced no curli fibers.

**Figure 4:**
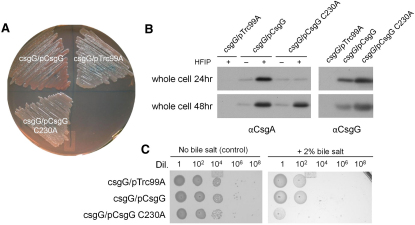
Functional Analysis of CsgG C230A (A) MC4100*csgG* harboring the empty vector, pCsgG and pCsgG C230A grown on YESCA-Congo red agar at 26°C for 24 hr. (B) Western blots of whole-cell lysates from a *csgG* mutant containing plasmid pCsgG, pCsgG C230A, or the empty vector grown on YESCA agar for 24 hr or 48 hr at 26°C. Bacteria were treated with (+) or without (−) HFIP before electrophoresis as indicated and probed with αCsgA antibody. Blots with non-HFIP treated samples were also probed with αCsgG antibody. (C) MC4100*csgG* expressing CsgG C230A was deficient in growth on bile salt agar. *csgG* harboring pTrc99A (the empty vector), pCsgG, and pCsgG C230A were inoculated in LB-no salt, normalized, and spotted on LB-no salt agar plates or LB-no salt agar with 2% (w/v) bile salt and incubated for 24 hr. See also Figure S8.

We also mutated the Cys230 residue to Ala, which is by far the most commonly observed residue occurring that position among homologs. The C230A mutant was expressed at a similar level to WT CsgG (Figure 4B, right panel). However, expression of CsgG C230A was not able to complement the Congo red binding by a *csgG* mutant under curli inducing conditions for 24 hr (Figure 4A). Moreover, when grown on YESCA agar for 24 hr, the C230A mutant produced only a small amount of bacteria-associated CsgA and interestingly this fiber subunit was present in an unpolymerized, SDS-soluble form (Figure 4B, left panel). This is in contrast with *csgG* expressing WT CsgG, which produced considerably more cell-associated CsgA and that required depolymerization by HFIP in order to migrate into the SDS-PAGE gel. Intriguingly after a further 24 hr incubation, the *csgG*/pCsgG C230A strain exhibited wild-type levels of cell-associated, SDS-insoluble CsgA. Because unpolymerized, SDS-soluble CsgA is found in the underlying agar of *csgG*/pCsgG C230A at 24 hr (data not shown), it appears that the delayed curli assembly phenotype is not likely due to the actual amount of CsgA being secreted, but rather some mishandling of the fiber subunit that causes a nucleation defect. These mutants therefore support our prediction that residues 226–245 of CsgG are likely to correspond to a region essential for oligomerization and pore-forming functionality.

### Structural Model of CsgG

Based on the proposition that CsgG possesses a single, C-terminal TM helix and by comparison with relevant known structures, we expect residues 16–225 to form the periplasmic domain, whereas 245–277 will be exposed at the cell surface. We therefore sought to obtain a homology model of the periplasmic domain. According to the PFAM database CsgG is found within the same clan as the N-terminal domain of TolB and the domain of unknown function (DUF) 330 (Finn et al., 2010). In agreement with this familial grouping, the two highest-scoring homology models for the periplasmic domain of CsgG created by PHYRE (Kelley and Sternberg, 2009) were based on the structures of these domains (PDB codes 2HQS and 2IQI). The homology model shows a loosely ordered N-terminal region, a pair of α helices packed against a β sheet (Figure 3A), which concurs with the predicted secondary structure of CsgG_16-226_ as well as circular dichroism data on CsgG-6xHis (Figure S7). Highly conserved residues within the CsgG family are found to cluster in the hydrophobic core of the model structure, lending support to our prediction. TolB is not known to oligomerize, however the DUF330 protein crystal contained an octameric ring on which we aligned models of CsgG into the oligomer shown in Figure 3B. In the homology model the overall diameter of the ring formed by the periplasmic domains is ∼100 Å and the TM pore is ∼20 Å, which is large enough to tolerate partially-folded polypeptides and consistent with a previous measurement by electron microscopy (Robinson et al., 2006). The periplasmic domains encircle a large central cavity that could accommodate subunits being secreted or CsgC, which we propose might modify the oxidation state of the C230 sites within the CsgG TM region.

**Figure 5:**
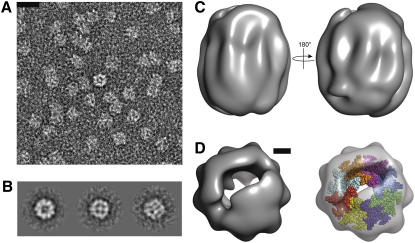
Negative-Stain EM Analysis of CsgG (A) Electron micrograph of CsgG at 50,000× zoom. Scale bar represents 20 nm. (B) Rotational averaging of two end views (left, center) and side view (right) of CsgG. (C) Initial low-resolution EM map of CsgG showing two side views. (D) Top view of low-resolution EM map showing the cavity running through the oligomer (left). Scale bar represents 20 Å. Manual docking of the octameric homology model of CsgG (spheres) into the EM map (right) indicates general agreement between the predicted and calculated models in terms of overall size and shape. See also Figure S5.

Finally, to test our predictions regarding CsgG structure and oligomerization we collected negative-stain electron microscopy data for CsgG-6xHis and performed image analysis and initial low-resolution structure calculations (Figure 5). Averaging of end views indicates pore-like structures and our octameric model of CsgG displayed in Figure 3 is consistent with overall and pore dimensions. Regions that could not be modeled, such as the N-terminal ∼30 residues, a periplasmic loop (residues 144–165) and extracellular domain (residues 246–277) along with bound detergent appear as additional electron density within Figure 5.

### The Functional Role of CsgC

**Figure 6:**
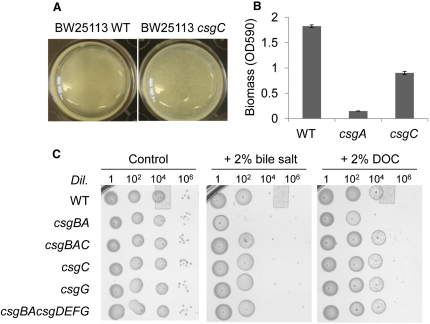
Functional Analysis of CsgC (A) Pellicle biofilm formation of BW25113 wild-type (WT) and *csgC* mutant. Strains were incubated in liquid LB-no salt at 26°C statically for 3 days. BW25113 formed a flat pellicle whereas *csgC* formed a wrinkled pellicle. (B) Biofilm quantification of MC4100 WT, *csgC* and *csgA* mutants. Strains were incubated in liquid YESCA at 26°C statically for 7 days and the biomass was quantified by crystal violet staining at OD_590_. (C) Bile salt sensitivity assays. Liquid *E. coli* cultures were inoculated at the dilutions (*Dil.*) indicated onto low salt LB agar plates with or agar with 2% (w/v) bile salt or 2% (w/v) deoxycholate and incubated at 26°C for 24 hr.

Our insights into the known and predicted structures of CsgC and CsgG, respectively, and the potential link between them prompted us to investigate further the effect CsgC, or loss thereof, has on curli biogenesis. The absence of *csgC* caused a morphological change of pellicle (air-water interface biofilm) formed by BW25113 (Figure 6A). A WT BW25113 strain formed a flat pellicle, whereas a BW25113 *csgC* mutant formed a wrinkled pellicle. In MC4100 strain background, mutation of *csgC* lead to a decrease in the amount of biofilm biomass attached to polystyrene surface (Figure 6B). It was previously reported that *Salmonella* strains lacking *agfC* were significantly more hydrophobic than wild-type, which may explain the altered biofilm characteristics we observed.

Next we discovered in bile salt sensitivity assays that CsgC influences the porosity of the subunit export complex. Gram-negative bacteria such as *E. coli* possess a degree of resistance to bile salts through active export mechanisms (Thanassi et al., 1997). However, we found that in the absence of curli formation, addition of the bile salt deoxycholate (DOC) to cells overexpressing CsgG arrests further growth (Figure S8). A similar effect was demonstrated previously using antibiotics (Robinson et al., 2006) and is presumably due to the influx of DOC through ungated CsgG pores. Co-expression of CsgC with CsgG made no significant difference to bile salt sensitivity. Conversely, cells overexpressing CsgE, CsgF and CsgG were only mildly affected by DOC suggesting that CsgE and/or CsgF assist in gating the pore. Interestingly, cells expressing the tripartite CsgEFG export complex alongside CsgC grew more slowly in the presence of bile salt. This implies that CsgC may have a role in increasing flux through CsgG.

To shed light on the role of CsgC in the context of endogenous curli formation we cultured wild-type and *csg* mutant strains under curli-permissive conditions in the absence and presence of a bile salt mixture or DOC. Wild-type cells were resistant to bile salts as was the *csgC* mutant (Figure 6C). Conversely, strains lacking the fiber subunit proteins CsgA and CsgB became sensitive. A logical explanation for this increase in sensitivity is that without cell-associated polymerization (Δ*csgBA*) there would be unrestricted access to the pore. Intriguingly, when *csgC* is subsequently knocked out as well as these fiber subunits (Δ*csgBAC*) then bile salt sensitivity reverts back to wild-type levels. Clearly the absence of CsgC results in an export complex that is closed to bile salt influx regardless of whether subunit proteins are available for export or not. In agreement with this, we observed that strains lacking *csgG* or *csgBA/csgDEFG* were as resistant to bile salt as wild-type, signifying that the fibers themselves are not the source of resistance, but rather it is the particular state of the subunit export complex formed by CsgG, with accessory proteins CsgE and CsgF. Thus it appears that the presence of CsgC increases the flux of macromolecules through CsgG, possibly in both directions, rendering the cell sensitive to bile salt unless it is also able to secrete both CsgA and CsgB and initiate fiber formation. These data are also consistent with a model of curli biogenesis in which CsgG forms a permanent base to the nascent fiber and not simply a subunit translocon because successful nucleation of the fiber, but not the fiber itself, has a protective effect against bile salt toxicity.

At the center of our hypothesis connecting CsgC with CsgG pore behavior are the co-conserved cysteine residues within each protein. We repeated the bile salt assay in a *csgG* mutant strain in which CsgG was supplied in *trans* from a plasmid. Cells containing empty vector or pCsgG showed equal resistance to bile salt (Figure 4C). When the transmembrane cysteine of CsgG (C230) was mutated to alanine the cells became sensitive to bile salt. At first glance, one might expect the C230A mutant to behave like the *csgC* mutant (i.e., resistant to bile salt) because removal of the key cysteine ought to abolish any interaction between the two proteins. Instead, the substitution of C230 by alanine may cause additional structural changes to the pore that promote influx of bile salt and provides further evidence to our overall hypothesis that C230 of CsgG influences pore behavior.

### Potential Redox Reactions Involving C230

It is tempting to speculate about the nature of the redox-related reactions occurring at C230 of CsgG and the role CsgC has in regulating these events. We do not yet understand why certain bacteria possess CsgC, however it clearly promotes their survival. One possibility is that CsgC creates or removes disulfide bonds that crosslink pairs of CsgG transmembrane helices and induce changes to pore characteristics, such as radius or selectivity. Although we have been unable to detect intermolecular disulfides within endogenous or recombinant CsgG, disulfide linkages can be induced both in vivo and in vitro by incubation with diamide (data not shown). This lends support to our structural model in which C230 residues are close in space within the oligomer. It is also possible that disulfide bonding only occurs naturally under certain environmental conditions, as is the case with the *E. coli* integral membrane transcriptional activator CadC (Tetsch et al., 2011).

Alternative possibilities are that the transmembrane cysteine of CsgG may form mixed disulfides with small-molecule thiols, or become oxidized to sulfenic acid during oxidative stress, or even become part of a sulfur-metal complex. There are reports of ion channels undergoing regulation by S-glutathionylation, however, to date there are no examples known to the authors in which this modification occurs in protein secretory systems (Aracena et al., 2003; Yang et al., 2011). However, additions to the C230 side chain could induce the observed changes in pore behavior and in this scheme CsgC would probably act as a reductant. In support of this concept, we made two intriguing observations regarding the structure of CsgG. First, from a topology perspective the periplasmic domain of CsgG is closely related to the archetypal thioredoxin (Trx) fold (Pedone et al., 2010). Trx family members are known to facilitate a variety of oxido-reductase reactions, such as introduction or removal of disulfide bonds, reversible oxidation of cysteines by small molecules, and detoxification of unwanted substances such as peroxides. At the center of these chemical reactions is a catalytic sequence motif, typically CxxC. In certain subgroupings of the Trx family, particularly the glutaredoxins, the latter cysteine is often substituted with serine (Atkinson and Babbitt, 2009). Furthermore, CxxS motifs are known to be conserved within redox-active proteins, often occurring at or just prior to the N-terminal end of an α-helix, but seldom found in other proteins (Fomenko and Gladyshev, 2002). It is interesting to note that the conserved cysteine of the TM helix of CsgG (C230) is actually part of a CxxS motif and it is located at the start of the helix. Furthermore, sequence alignment of CsgG homologs from a broad range of bacterial species show that the CxxS motif is completely conserved among the few bacteria that possess a *csgC* gene, whereas a large majority of other species display an Axxx sequence (data not shown). Thus there is a distinct possibility that S233 promotes the reactivity of C230 toward its physiological substrate.

Our work provides the first atomic-resolution insight into the proteins that mediate curli fiber subunit translocation across the outer-membrane. The high resolution structure of the accessory protein CsgC revealed a redox-active CxC motif that we suggest regulates the redox status of C230 of CsgG. The purpose of this system may be to fine-tune amyloid fiber formation to improve cellular fitness during certain environmental conditions. The absence of this system within the majority of curli-producing bacteria indicates that CsgC provides an additional level of control over the complex phenomenon of curli biogenesis. Future research into the structure and function of CsgG should enlighten further this fascinating protein export system.

## Experimental Procedures

### CsgC Sample Preparation, Crystallization, and Structure Refinement

CsgC expression, purification, and crystallization, as well as data collection and processing have been described elsewhere (Salgado et al., 2011). Initial phases for both native crystal forms 1 and 2 (reduced and oxidized CsgC, respectively) were calculated by molecular replacement with Phaser (McCoy et al., 2007), using the previously described CsgC SeCys/SeMet model (PDB code 2XSK) (Salgado et al., 2011), within the CCP4 program suite (CCP4, 1994), in order to obtain interpretable electron density maps. Iterative cycles of model building and refinement were carried out using COOT (Emsley and Cowtan, 2004) and REFMAC5 (Vagin et al., 2004) respectively, to determine the final reduced and oxidized CsgC models, deposited within the PDB as 2Y2T and 2Y2Y, respectively. Final model refinement statistics are detailed in Table 1.

### CsgG-6xHis Sample Preparation

The *csgG* gene was amplified from *E. coli* O157:H7 genomic DNA and the product digested with *Nco*I and *Xho*I (Fermentas) and ligated into pET28 (Novagen), in-frame with a C-terminal histidine tag. CsgG-6xHis was expressed in C41 (DE3) cells cultured in 3 L Terrific Broth. Cultures were grown at 37°C with shaking until mid-log phase, cooled on ice, and incubated at 18°C for 15 min. Expression was induced by 0.2 mM IPTG and the cells harvested after 16 hr by centrifugation, resuspended in ∼300 ml lysis buffer (20 mM Tris-HCl pH 8.0, 2 mM MgCl_2_, 10 U Benzonase nuclease [Novagen], and protease inhibitors [PMSF, Aprotinin, Pepstatin, Leupeptin]) and lysed by a cell disruptor (Constant Systems, 25 kpsi). Unbroken cells/debris were removed by centrifugation at 5000 × g. Membranes were separated from soluble material by ultracentrifugation (Beckman Optima L-100 XP) at 41,000 rpm for 90 min. The pellet was resuspended in 180 ml 20 mM Tris-HCl pH 8.0, 0.5% (w/v) *N*-Lauroylsarcosine sodium salt by stirring at 4°C for 60 min. The outer-membrane fraction was then obtained by ultracentrifugation for 60 min. CsgG-6xHis was solubilized from the pellet by overnight stirring in 100 ml 20 mM Tris-HCl pH 8.0, 150 mM NaCl, 2% w/v *n*-dodecyl-β-D-maltoside (DDM) at 4°C. Insoluble material was removed by ultracentrifugation for 30 min. Imidazole was added to 40 mM and the sample rocked with 2 ml Ni-NTA resin (QIAGEN) for 60 min. The resin was drained and washed with 30 ml 20 mM Tris-HCl pH 8.0, 150 mM NaCl, 60 mM imidazole, 1 mM DDM. CsgG-6xHis was eluted from the column by addition of 20 mM Tris-HCl, 150 mM NaCl, 300 mM imidazole, and 0.54 mM DDM. The sample was purified further using a Superdex 200 16/60 size-exclusion column equilibrated in 20 mM Tris-HCl pH 8.0, 150 mM NaCl, and 0.54 mM DDM.

### Negative Stain Electron Microscopy

A 2-μl sample of CsgG-6xHis at ∼50 μg/ml was applied to glow-discharged continuous-carbon-coated copper grids (Agar Scientific, UK), washed with 30 μl 2% (w/v) uranyl acetate, blotted, and air-dried. Microscopy was performed using a Philips CM200 FEG electron microscope. Images were collected on a 16-megapixel CCD camera (TVIPS GMBH) and a pixel spacing of 1.76 Å, as measured on the specimen plane. A total of 12,301 particles were picked interactively using the Boxer program from the Eman package. All subsequent image processing, except alignments, were performed using Imagic (ImageScience GmbH). Alignments were performed using the “timalign” program from the Tigris (http://sourceforge.net/projects/tigris) package. The particles were subjected to an initial centering followed by classification. Class-averages that were considered to be of “good” quality by eye were assigned random Euler angles. These class-averages were used to obtain a 3D reconstruction, which was then forward projected in order to create references for the next round of alignment. This was followed by classification, Euler angle assignment and 3D reconstruction. This procedure was repeated over 20 times until the changes in the 3D reconstruction were marginal. The whole point of starting with random Euler angle assignment was to avoid reference bias. In order to reinforce the validity of the model derived, we started up with an alternative initial random Euler angle assignment. This yielded a model similar to the original one and the model shown is an average of the two.

### Mass Spectrometry

Mass analysis of CsgC was performed by Dr. James Ault (Mass Spectrometry Facility, Astbury Centre for Structural Molecular Biology, University of Leeds, UK) using electrospray ionization and a Q-TOF mass spectrometer.

### Measurement of Reduction Potential

CsgC was oxidized or reduced by incubation for 1 hr at 293 K with 50 mM GSSG or 10 mM TCEP, respectively, and then purified by gel filtration. The reduction potential of CsgC was calculated as described previously (Derewenda et al., 2009). Data were fitted by nonlinear least-square methods within ORIGIN (OriginLab) to the Nernst equation with the reduction potential of glutathione set as −234 mV at 293 K (Veine et al., 1998).

### Model of CsgG

The transmembrane (TM) helix of CsgG was located by combining results from TMpred, MPex, hydrophobic cluster analysis, and secondary structure predictions (Callebaut et al., 1997; McGuffin et al., 2000; Snider et al., 2009). The TM helix was modeled by mutating the equivalent region in Wza using Coot (Emsley and Cowtan, 2004) followed by energy minimization by GROMACS using the NOMAD-REF server (http://lorentz.dynstr.pasteur.fr). PHYRE was used to identify homologs of CsgG_16-226_ and create the homology models of the periplasmic domain based on structures of TolB and DUF330 (Kelley and Sternberg, 2009). To create the model of the CsgG octamer shown in Figure 3, TolB-derived CsgG monomers were individually aligned to the DUF330 monomers using CEalign (Shindyalov and Bourne, 1998).

### Bacterial Strains and Plasmids

*E. coli* MC4100 strains were used for the bile salt assay, biofilm quantification and western blotting. BW25113 strains were used for pellicle biofilm assay. MC4100*ΔcsgC*, MC4100*ΔcsgBAC* were constructed according to the methods described previously (Datsenko and Wanner, 2000). MC4100*ΔcsgC* and MC4100*csgΔBAC* were constructed by deleting *csgC* gene and the *csgBAC* operon from wild-type MC4100, respectively. Linear PCR fragments were amplified by using plasmid pKD4 as the template and the primer sets **TATTACAGAAACAGGGCGCAAGCCCTGTTTTTTTTCGGGAGAAGAATATG**GTGTAGGCTGCAGCTGCTTC, **ATTCATCTTATGCTCGATATTTCAACAAATTAAGACTTTTCTGAAGAGGG**CATATGAATATCCTCCTTA to make MC4100*csgC::kan* and **GAAATGATTTAATTTCTTAAATGTACGACCAGGTCCAGGGTGACAACATG**GTGTAGGCTGGAGCTGCTTC, **ATTCATCTTATGCTCGATATTTCAACAAATTAAGACTTTTCTGAAGAGGG**CATATGAATATCCTCCTTAG to make MC4100*csgBAC::kan* (bold text represents sequence complementary to *csg* genes). The kanamycin cassette was eliminated by introducing the temperature sensitive vector pCP20 that expresses the FLP recombinase and subsequent incubation at 42°C to remove pCP20. The BW25113*ΔcsgC* was made by flipping the kanamycin cassette out from BW25113*Δcsg::kan* from the Keio collection (Baba et al., 2006). Gene deletion was confirmed by the *csgB* upstream primer CACGGCTTGTGCGCAAGACA and *csgC* flanking primers AGTGGAACGGCAAAAATTCTG and TTATTCTATTTCCTCAATGA.

To make the plasmid expressing CsgG C230A, the fragment of CsgG C230A was amplified by fusion PCR with the primer set: CATGCCATGGCCATGCAGCGCTTATTTCTTTTGGTTGCCG, CCTGTTATGCTGGCGCTGATGTCGGCT, AGCCGACATCAGCGCCAGCATAACAGG, CGGGATCCTCAGGATTCCGGTGGAACCGACATATG (the restriction sites are underlined). The fragment was cloned into the *Nco*I and BamHI sites of the pTrc99A vector.

### Bile Salt and Deoxycholate Assay

Overnight cultures in LB broth were normalized by optical density at 600 nm. Serial base two dilutions were prepared and 3 μl of diluted culture were spotted on LB-no salt agar (5 g/L yeast extract, 10 g/L bacto tryptone, and 1.5% [w/v] agar) plates containing 2% (w/v) bile salt (Sigma) or 2% (w/v) deoxycholate (DOC) to induce curli expression. Bacteria were cultured at 26°C for 24 hr before visual assessment of bacterial growth.

### Pellicle Formation Assay

Overnight cultures in LB broth were normalized by optical density at 600 nm. A 2-μl aliquot of bacterial culture was diluted into 2 ml liquid LB-no salt broth in wells of a 24-well cell culture microtiter plate (Greiner Bio-one) (Cegelski et al., 2009). Pellicle formation was observed after 3 days static growth at 26°C.

### Biofilm Quantification

MC4100 strains form an air-liquid-interface biofilm on the polystyrene wall of 96-well plates. Quantification of biomass was performed as described previously (Merritt et al., 2005). Tested strains were grown overnight at 37°C in LB, normalized by OD_600_, and diluted by 1:100-fold into 200 μl liquid LB-no salt or YESCA, incubated at 26°C statically for 5 or 7 days. Six replicates of each strain were inoculated. Planktonic bacteria were removed and plates were washed three times with deionized water. Biofilm was stained with 200 μl 0.1% crystal violet (CV) for 5 min at room temperature. The plates were then rinsed three times with water. The biomass was determined by solubilize CV into 95% ethanol and measure the OD at 590 nm.

### Western Blotting

CsgA and CsgG levels were determined by western blotting of bacterial whole-cell lysates as previously described (Wang and Chapman, 2008). Bacteria grown on YESCA agar plates at 26°C for 24 hr or 48 hr were collected in PBS buffer, normalized by OD_600_, and pretreated with or without HFIP (1,1,1,3,3,3-Hexafluoro-2-propanol). Samples were separated by 15% SDS-PAGE gel and proteins were probed with αCsgA or αCsgG antibody.

## Figures and Tables

**Table 1:** Crystallographic data and refinement statistics for CsgC

	Oxidized	Reduced
Data collection and processing statistics		
X-ray source	In-house	PROXIMA1, SOLEIL
Wavelength (Å)	1.5418	1.1271
Space group	C222_1_	C2
Unit cell (a, b, c [Å]; β[°])	37.0, 80.5, 70.6; 90.0	79.49, 36.43, 39.52; 117.45
Resolution range (Å)	20.0–2.40 (2.45–2.40)	33.80–2.39 (2.45–2.39)
No. of images	226 (1° oscillation)	360 (1° oscillation)
Observations total/unique	18,583/4326	28,097/7646
Redundancy	4.3	3.7
Completeness (%)	100.0 (100.0)	97.0 (75.4)
I/σI	23.2 (6.2)	18.4 (7.8)
R_merge_ (%)	5.3 (15.8)	5.3 (15.8)
R_meas_ (%)	—	6.0 (18.6)
Molecules in A.U.	1	1
Solvent content (%)	41.0	41.0
Refinement statistics		
R^a^	0.265	0.239
R_free_^b^	0.328 (339 reflections)	0.273 (226 reflections)
No. atoms		
Total	746	748
Protein	729	729
Water	9	19
Other	8	—
Average B (overall)	20.9	26.5
Rmsd		
Bond length	0.021	0.021
Bond angles	2.049	2.103
Ramachandran plot statistics (% residues)^c^		
Most favored	97.8	97.8
Allowed	2.2	2.2
Disallowed	0.0	0.0
PDB accession code	2Y2Y	2Y2T

PDB, Protein Data Bank; Rmsd, root-mean-square deviation.

a $R=\sum_{hkl}\left|\left|F_{obs}\right|-\left|F_{calc}\right|\right|/\sum_{hkl}\left|F_{obs}\right|$.

b R_free_ was calculated from a randomly selected 5% of unique reflections that were omitted from structure refinement.

c Determined by MolProbity (http://kinemage.biochem.duke.edu).

## Supplemental Information

Document S1. One Table, Eight Figures, and Supplemental Experimental Procedures
