# Supplementary material for: Atomic Resolution Insights into Curli Fiber Biogenesis
Source: Structure. 2011 Sep 7;19(9-16):1307–16. doi: 10.1016/j.str.2011.05.015 (PMC3173608; doi:10.1016/j.str.2011.05.015)
Supplement: Document S1. One Table, Eight Figures, and Supplemental Experimental Procedures [file mmc1.pdf]

Structure *19*

Supplemental Information

Atomic Resolution Insights

into Curli Fiber Biogenesis

Jonathan D. Taylor, Yizhou Zhou, Paula S. Salgado, Ardan Patwardhan, Matt McGuffie, Tillmann Pape, Grzegorz Grabe, Elisabeth Ashman, Sean C. Constable, Peter J. Simpson, Wei-chao Lee, Ernesto Cota, Matthew R. Chapman, and Steve J. Matthews

Supplementary Information Inventory

Supplementary Table

Table S1. Statistical checks of oxidised CsgC crystallographic data, related to Table 1.

Supplementary Figures

Figure S1. NMR relaxation analysis of CsgC, related to Figure 1.

Figure S2. Alignment of the *csgC* and *dsbD* DNA sequences, related to Figure 2.

Figure S3. Assigned ^{15}N HSQC of CsgC, related to Figure 1.

Figure S4. Redox analysis of CsgC, related to Figure 2.

Figure S5. Oligomerisation state of CsgG, related to Figures 3 and 5.

Figure S6. Bioinformatic analysis of CsgG, related to Figure 3.

Figure S7. Circular dichroism spectrum of CsgG, related to Figure 3.

Figure S8. Solution state bile salt sensitivity assay, related to Figure 4.

Supplemental Experimental Procedures

Supplementary References

	Indexed in $C222_1$	Indexed in P1
**POINTLESS**		
Identified space group	$C222_1$	$C222_1$
Laue group probability:	1.000	0.993
Systematic absence probability:	0.996	0.983
Total probability:	0.996	0.976
Space group confidence:	0.994	0.968
Laue group confidence	1.000	0.991
**TRUNCATE**		
Twinning detected?	no	L test: none<br>H test: 0.3 fraction (due to misindexing in P1, operators relate to $C222_1$ reindexing)
<E> (Expected = 0.886, Perfect Twin = 0.94)	0.866	0.876
<E**3> (Expected = 1.329, Perfect Twin = 1.175)	1.417	1.406
<E**4> (Expected = 2, Perfect Twin = 1.5)	2.338	2.383
**Phenix.triage**		
<I^2>/<I>^2	2.291	2.234
<F^2>/<F>^2	0.751	0.764
<|E^2-1|>	0.785	0.766
<L>,<L^2>	0.505, 0.344	0.493, 0.329
Multivariate Z-score L-test	4.090	2.179
H test	-	0.4 fraction (3 operators due to misindeixing in P1)
Max deviation acentric	0.052	0.026
Max deviation centric	0.021	0.683
<Nzobs- NZtwinned)>_acentric	+0.036	+0.019
<Nzobs- NZtwinned)>_centric	-0.001	-0.467
Pseudo-translation detected	none	none
Patterson peak off-origin (relative height)	9.62	11.67
P-value (height) (<0.05 = weak pseudotranslation)	0.1825	0.08942

Table S1, related to Table 1. Statistical checks of oxidised CsgC crystallographic data. Due to the relatively high free R factor for the oxidised form the original data were processed and analysed in both the C222 and P1 space groups. POINTLESS, TRUNCATE and Phenix.triage analyses were performed to search for twinning and pseudosymmetry within the crystal lattice. The results indicate the absence of these complicating issues and support assignment of the C222 space group.

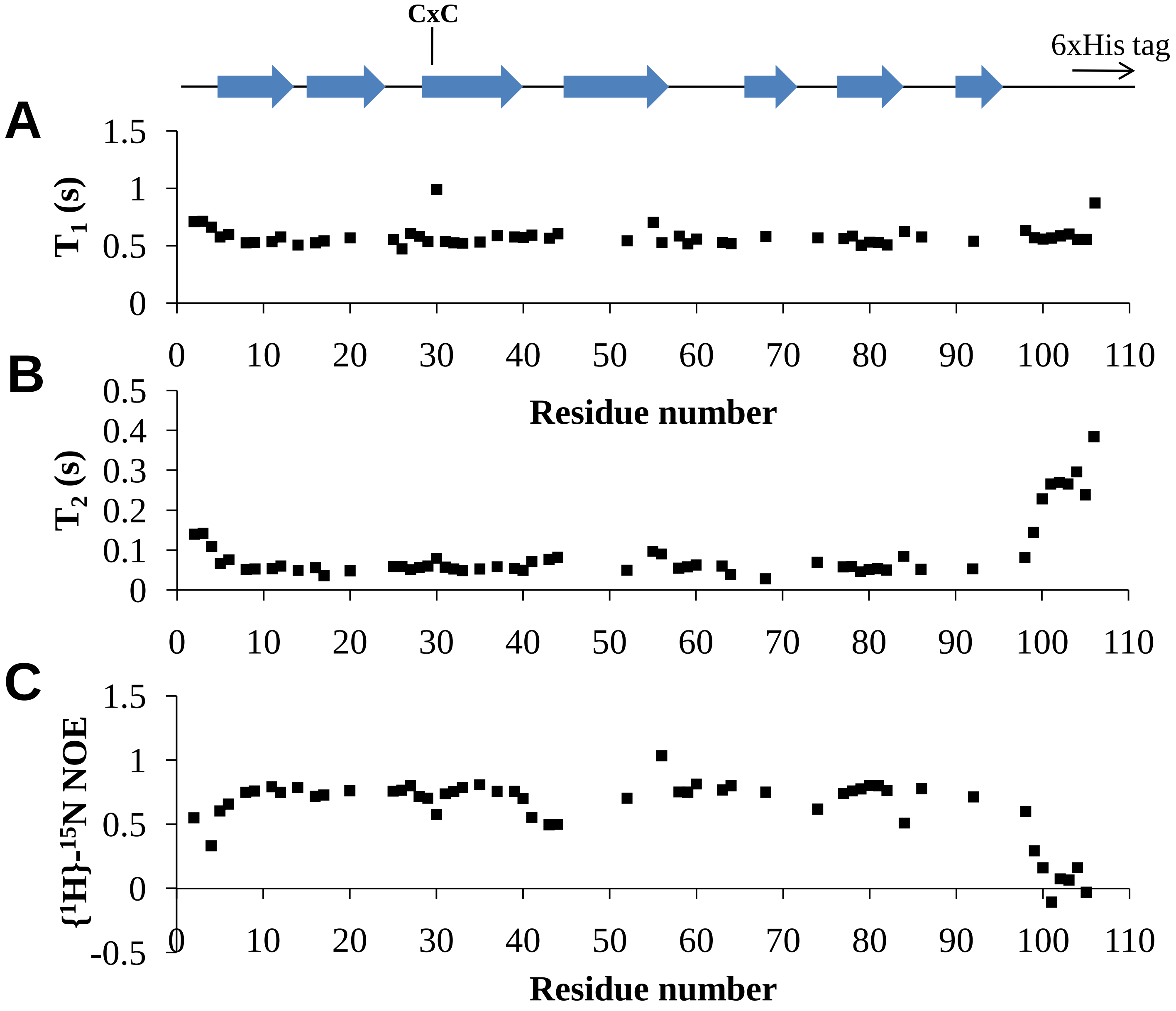

Figure S1, related to Figure 1. Dynamic properties of oxidised CsgC backbone amides. (**A**) ^{15}N longitudinal relaxation time (T_1). (**B**) ^{15}N transverse relaxation time (T_2). (**C**) $\{^1H\}$-^{15}N heteronuclear NOE value. Data points from peaks that overlap are excluded from analysis. The β-strands (solid arrows), the position of CXC motif and 6xHis tag are indicated for interpretation of the relaxation data. CsgC is generally well ordered apart from the extreme N and C-termini and the 6xHis tag, consistent with the absence of electron density for these regions.

```
             D  Q  A  F  A  F  D  F  Q  Q  N  Q  H  D  L  N  L  T  W  Q
nDsbD       GATCAAGCCTTTGCTTTTGATTTTCAGCAAAACCAACATGACCTTAATCTGACCTGGCAG 60
                        **  **    * ***  * *  ***    * *****  ****  ***
CsgC        ------------GCACTT----TCCAGTCAGAT-AAC----CTTTAATACGACCCAGCAA 39
                         A  L      S  S  Q  I   T      F  N  T  T  Q  Q

             I  K  D  G  Y  Y  L  Y   R  K  Q  I  R  I  T  P  E  H  A  K
nDsbD       ATCAAAGACGGTTACTACCTCTAC-CGTAAACAGATCCGCATTACGCCGGAACACGCGAA 119
                  ** *  ** ****  **  * * **    **    **** *           **
CsgC        ---GGGGATGTGTA-TACCATTATTCCTGAA---GTCACTCTTACTC-----------AA 81
                G  D  V  Y   T  I  I  P  E     V  T  L  T             Q

              I  A  D  V  Q  L  P  Q  G  V  W   H  E  D  E  F  Y  G  K
nDsbD       AATTGCCGACGTGCAGCTGCCGCAAGGCGTCTG-GCATGAAGATGAGTTTTACGGCAAAA 178
              *** *   ******    *  *    ***   **  ****      ** * ******
CsgC        TCTTGTC--TGTGCAGAGTACAAATATTGTCCCTGCGCGAAGGCAG--TTCAGGGCAAAG 137
             S  C    L  C  R  V  Q  I  L  S  L  R  E  G  S    S  G  Q  S

            S  E  I  Y  R  D  R  L  T  L  P  V  T  I  N  Q  A  S  A  G
nDsbD       GCGAGATTTACCGCGATCGGCTGACGCTTCCCGTCACCATCAACCAGGCGAGTGCGGGAG 238
             * ***   * *  **      *** *** *  *  *    ** **  * * ***
CsgC        TC-AGACGAAGCAAGA---AAAGACCCTTTCATTGCCTGCTAATCAACCCATTGCTTTGA 193
               Q  T  K  Q  E     K  T  L  S  L  P  A  N  Q  P  I  A  L

            A  T  L  T  V     T  Y  Q  G  C    A  D  A  G  F  C  Y  P  P
nDsbD       CGACGTTAACTGT---CACCTACCAGGGCTGT--GCTGATGCCGGTTTCTGTTATCCGCC 293
            *** *** * * *    *  * ** ** *  *  * ***     ***  *  * *
CsgC        CGAAGTTGAGTTTAAATATTTCCCCGGACGATCGGGTGAAAATAGTTGTTACTGTTTCTG 253
            T  K  L  S  L  N  I  S  P  D  D  R  V  K  I  V  V  T  V  S

              E  T  K  T  V  P  L  S  E  V  V  A  N  N  A  A  P  Q  P  V
nDsbD       AGAAACCAAAAACCGTTCCGTTAAGCGAAGTGGTCGCCAACAACGCAGCGCCACAGCCTGT 353
            *   **   *  *  * * ** * *           ****** *    *** *
CsgC        ATGGAC---AGTCACTTCATTTATCA----------CAACAATG----GCCGC------- 289
            D  G     Q  S  L  H  L  S            Q  Q  W      P

              S  V  P  Q  Q  E  Q  P  T  A  Q
nDsbD       GTCTGTTCCGCAGCAAGAGCAGCCCACCGCGCAA 387
              ** ***       ***  ** *
CsgC        -CCTCTTC-------AGAAAAGTCT--------- 306
             P  S  S         E  K  S
```

Figure S2, related to Figure 2. Sequence alignment of *csgC* and *nDsbD*. The translated sequences of nDsbD (blue) and CsgC (green) are shown above and below the DNA sequence alignment. Alternate codons are shaded in grey to highlight regions where sequences are similar, yet translated in a different reading frame. This suggests a common ancestry. The codons that give rise to C29 and C31 in CsgC (TGT and TGC, highlighted in red) are present in nDsbD but are translated differently due to nucleotide insertions**.** Alignment was performed by ClustalW (www.ebi.ac.uk/Tools/msa/clustalw2).

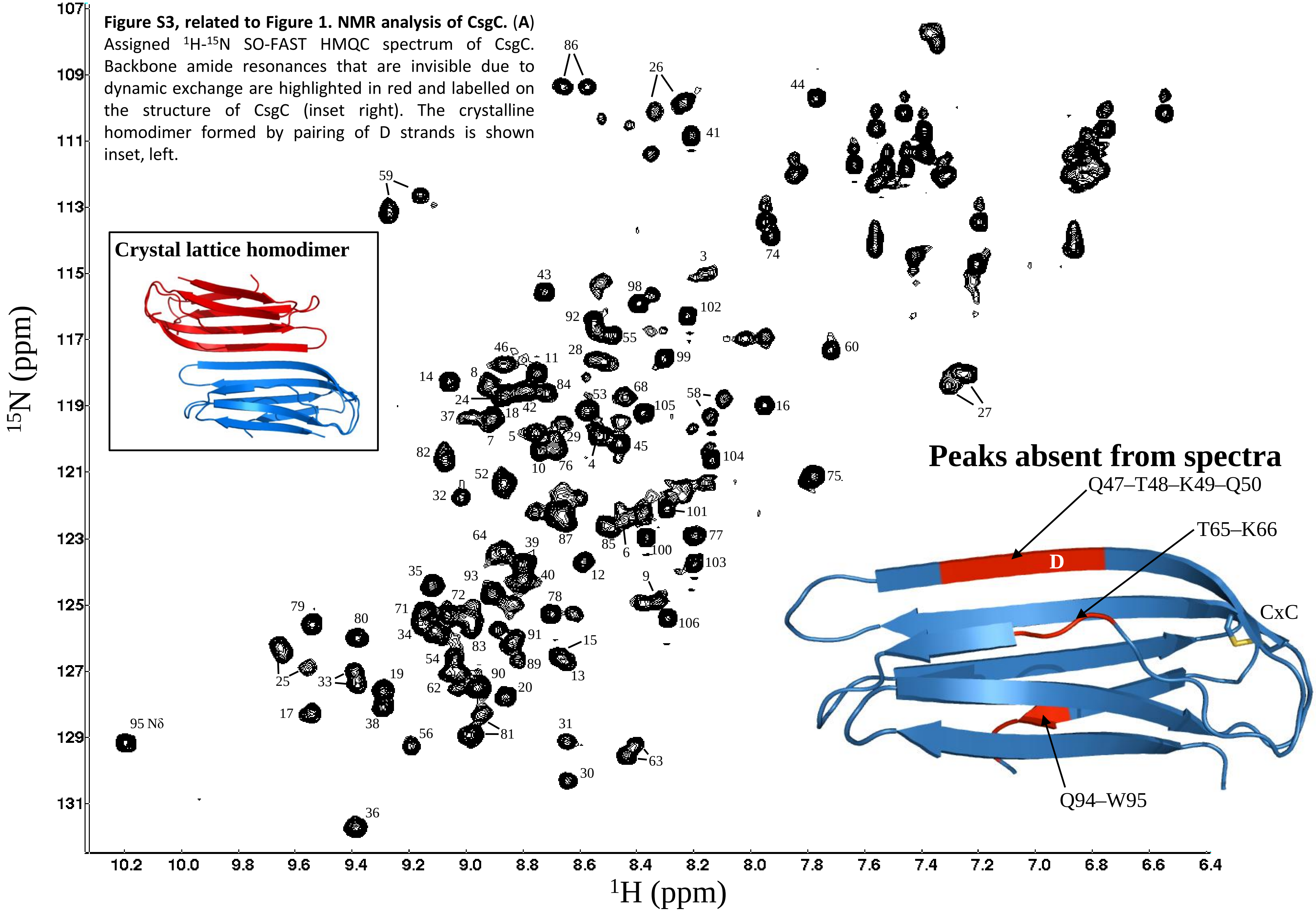

Figure S3, related to Figure 1. NMR analysis of CsgC. (A) Assigned ^{1}H-^{15}N SO-FAST HMQC spectrum of CsgC. Backbone amide resonances that are invisible due to dynamic exchange are highlighted in red and labelled on the structure of CsgC (inset right). The crystalline homodimer formed by pairing of D strands is shown inset, left.

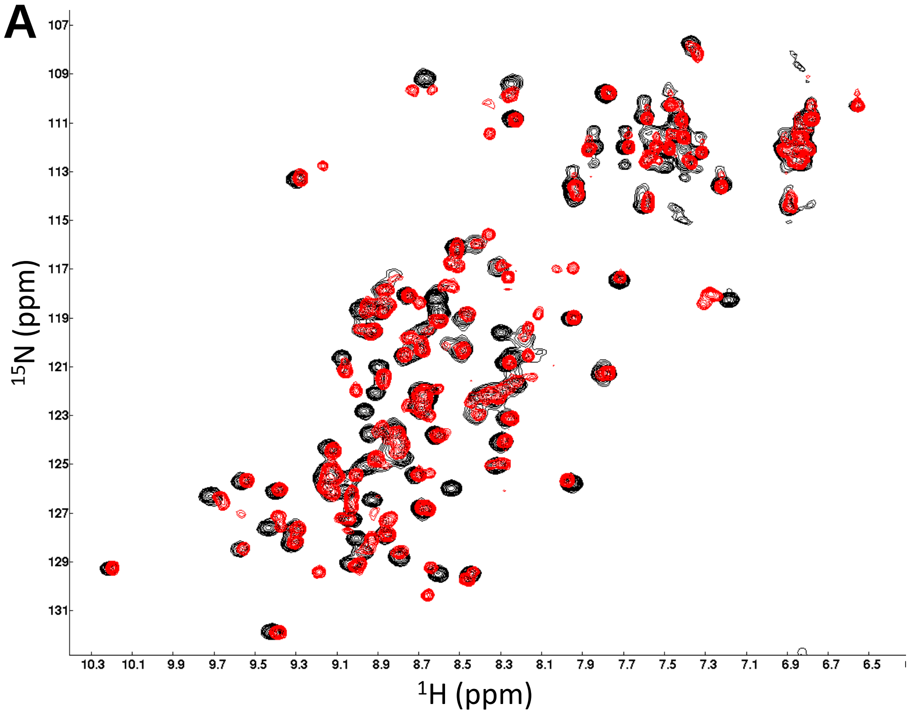

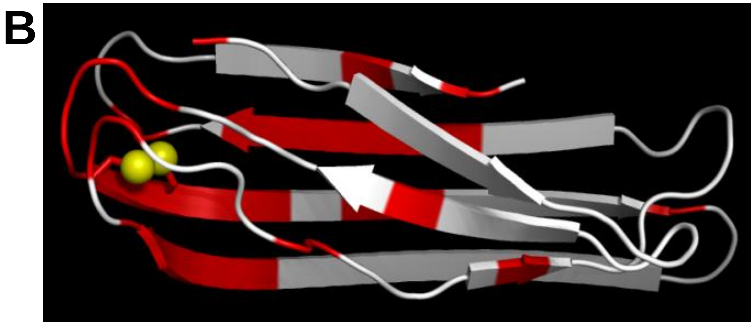

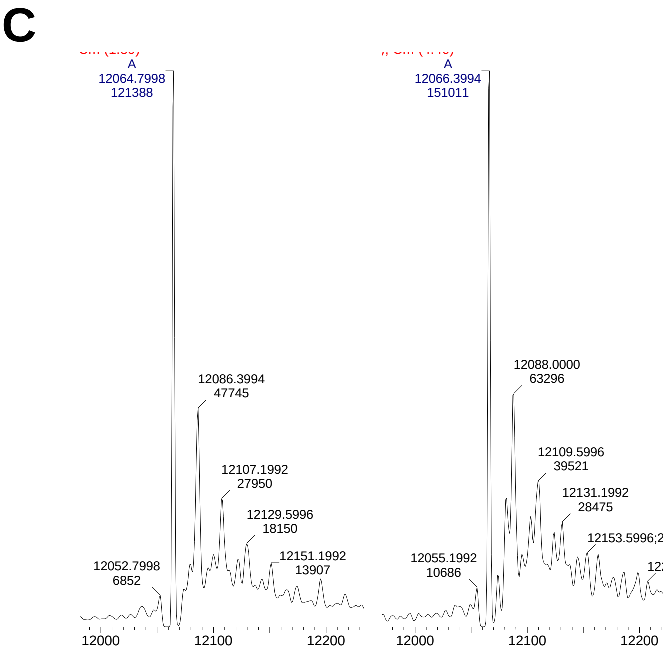

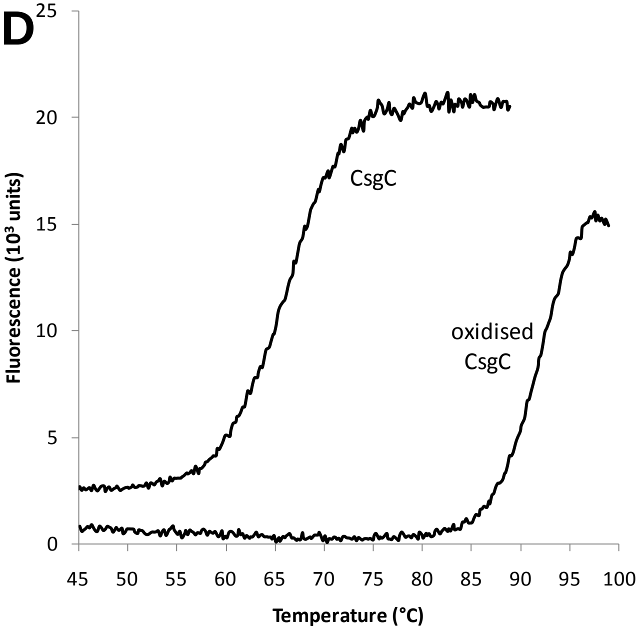

Figure S4, related to Figure 2. Effect of redox state of CsgC. (**A**) Comparison of HSQC spectra of the reduced (red) and oxidised (black) CsgC. (**B**) Amides that show significant chemical shift changes are highlighted in red on the CsgC structure. The sulphur atoms of C29/C31 are represented as yellow spheres. (**C**) Electrospray ionisation mass spectroscopy of oxidised (left) *vs*. reduced (right) CsgC. (**D**) Thermal melting of CsgC. The fluorescence of SYPRO-orange dye was measured during temperature ramping. Increasing emission corresponds to exposure of hydrophobic surface through unfolding.

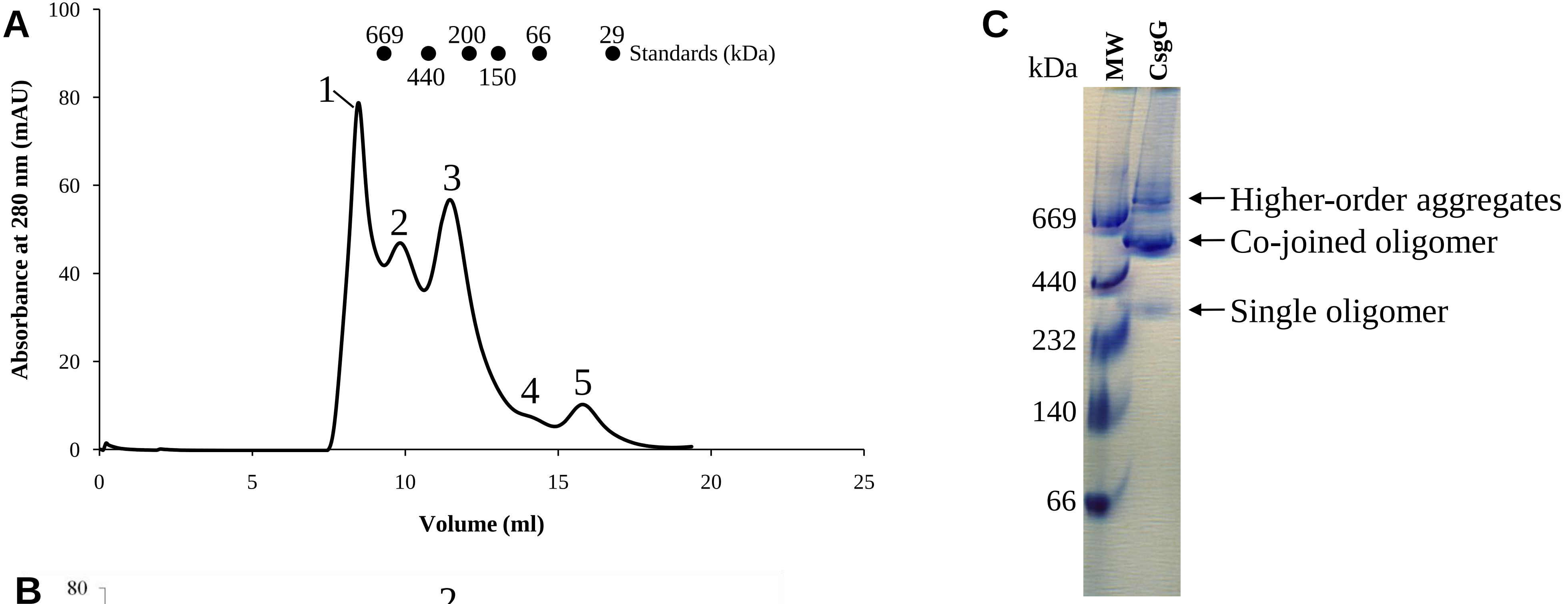

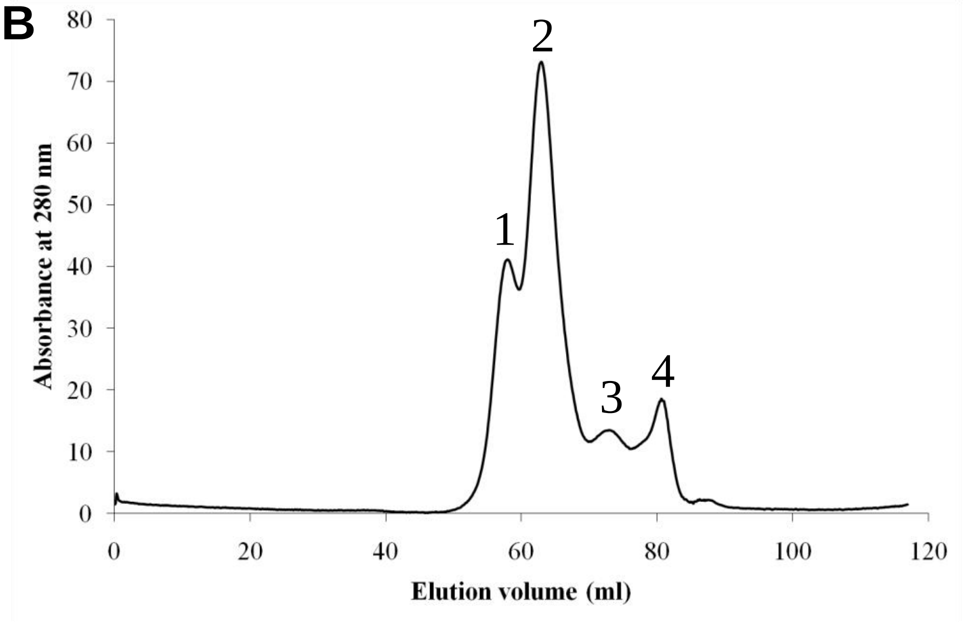

Figure S5, related to Figures 3 and 5. Estimation of CsgG oligomeric stoichiometry. (**A**) Analytical gel filtration chromatography of purified CsgG$_{16\text{-}277}$. The non-detergent-solubilised oligomeric form is ~250 kDa (peak 3), suggestive of an octameric complex. It also self-associates into co-joined oligomers (2) and higher-order aggregates (1) and dissociates into dimers (4) and monomers (5). (**B**) Analytical gel filtration of detergent-solublised CsgG-6xHis. The protein-detergent complex (PDC) runs at ~330 kDa (peak 2). DDM micelles usually contribute ~85-100 kDa to the PDC, consistent with a protein component of ~240 kDa – i.e. an octamer. However due to hydrodynamic effects caused the detergent and the hollow pore, this value should be treated with caution. The PDC also show self-association (peak 1) and instability (peaks 3 and 4). (**C**) Blue Native PAGE analysis of CsgG-6xHis. The protein migrates as several species, consisting of the single oligomer, a co-joined oligomer, and higher order aggregates of the oligomer. Whilst accurate determination of the mass of membrane proteins is difficult using this approach, the result is consistent with gel filtration and observations of stoichiometry from our EM analysis (Figure 5).

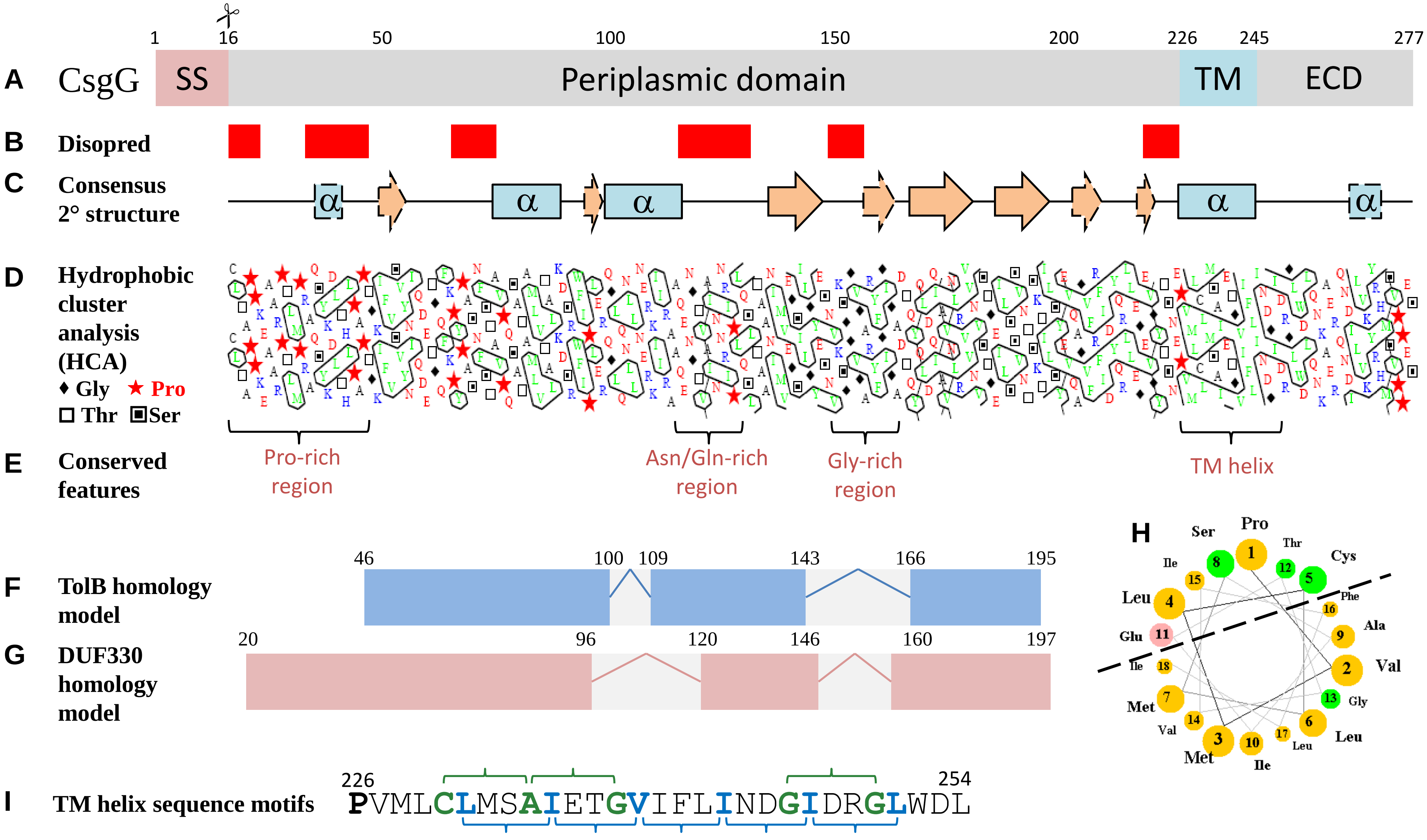

Figure S6, related to Figure 3. Bioinformatic analysis of CsgG. (**A**) The location of the transmembrane (TM) helix, extracellular domain (ECD), periplasmic domain and signal sequence (SS) CsgG is shown. (**B**) Prediction of disordered regions by Disopred. (**C**) Consensus secondary structure prediction by PHYRE. Dashed lines indicate relatively weak prediction confidence. (**D**) Hydrophobic cluster analysis (HCA) reveals interesting sequence patterns that reflect secondary structure, linkers, domain boundaries and regions rich in particular residues. (**E**) HCA features conserved amongst CsgG homlogues. (**F**) and (**G**) Regions included in the TolB- and DUF330-based homology models of CsgG, respectively. (**H**) Helical wheel projection of the predicted TM helix (226-245). The dashed line segregates the hydrophobic surface (below line) from the hydrophilic surface (above the line). Figure generated using Helical Wheel Applet. (**I**) Sequence of TM region indicating contiguous helix-helix interaction motifs: [GAC]-xxx-[GAC] (green) and [IV]xxx[IVL] (blue). Proline is also favoured at the N-terminal position within TM helices.

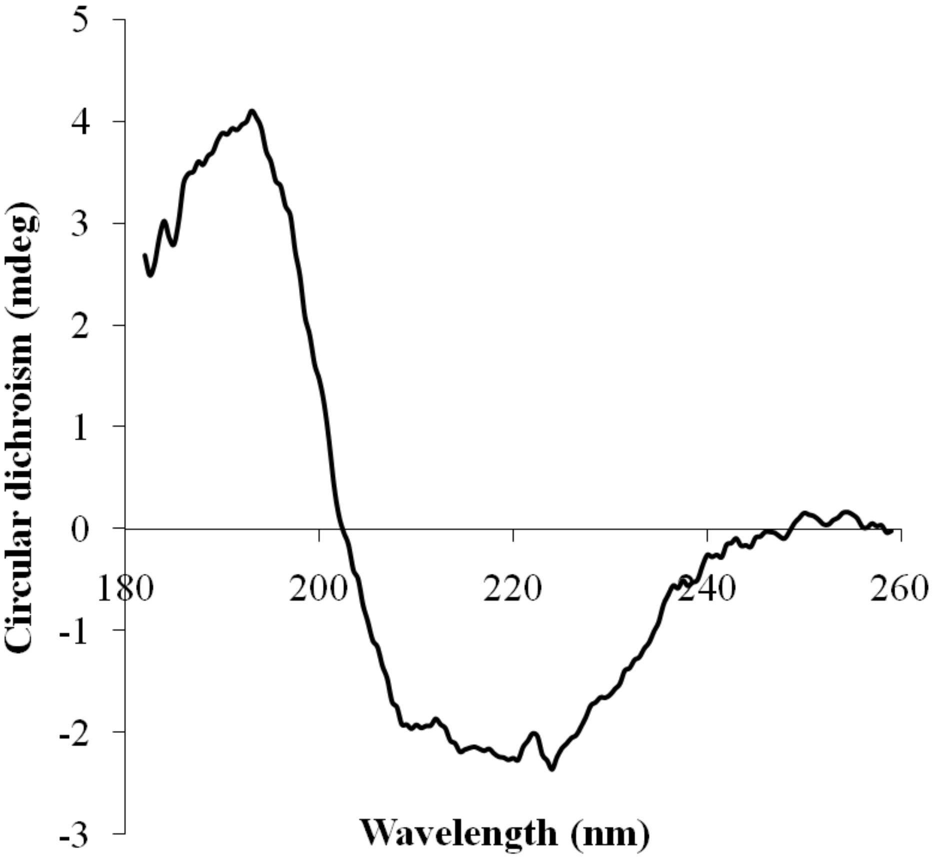

Figure S7, related to Figure 3. Circular dichroism analysis of CsgG-6xHis. The spectrum of CsgG is characteristic of a mixed alpha/beta fold, as opposed to the normal β-barrel observed in outer-membrane proteins, which supports our homology model structure prediction.

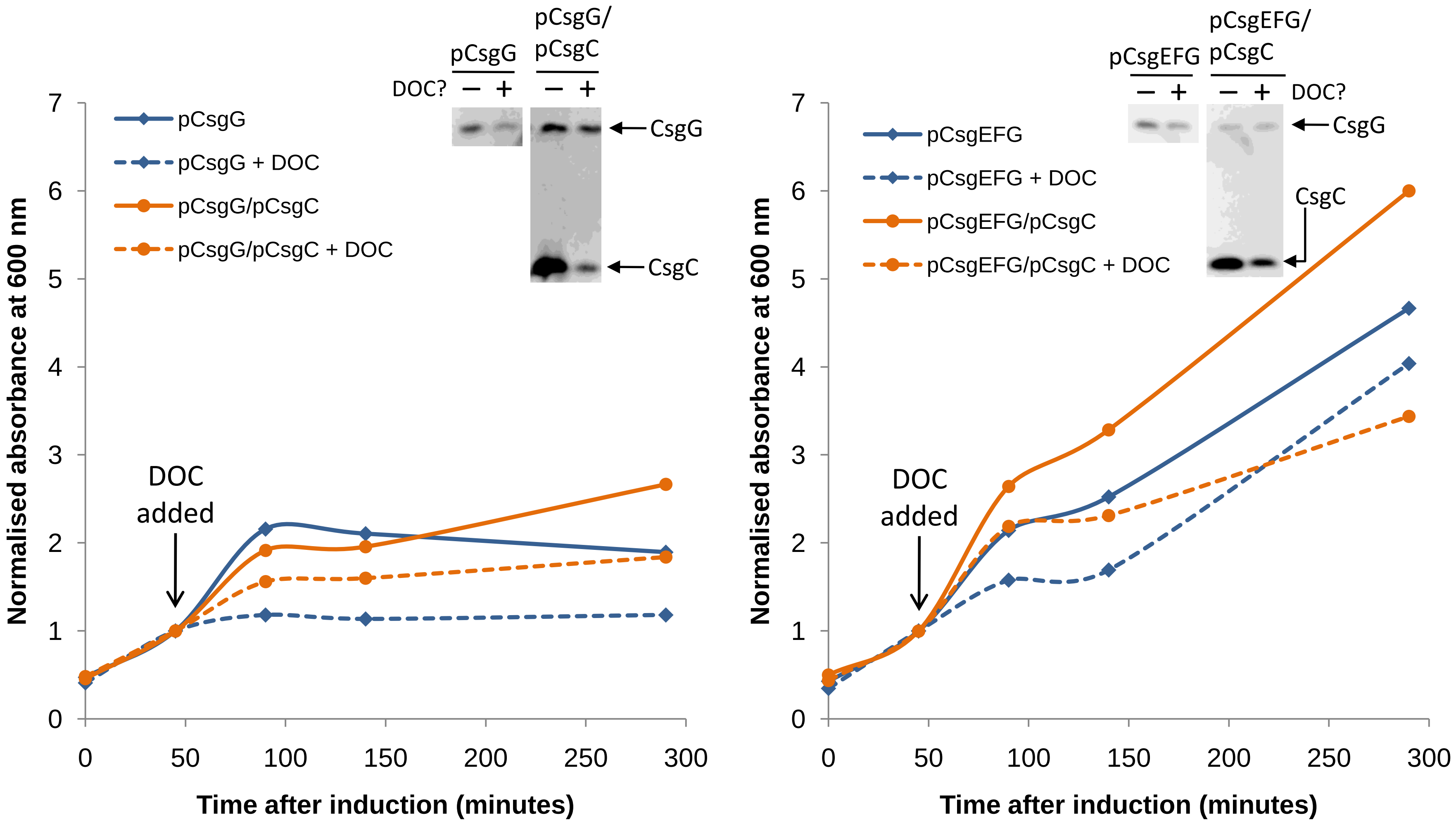

Figure S8, related to Figure 4. Liquid-phase bile salt sensitivity assay. The ability of *E. coli* C41 (DE3) cells to grow in the absence or presence of deoxycholate whilst overexpressing recombinant curli proteins was tested. Cell cultures were grown to ~0.5 OD_{600} units at which point (co-)expression of the Csg proteins indicated were induced (t=0). After 45 minutes, deoxycholate (DOC) was added and cell growth was monitored for several hours. To aid clarity, absorbance readings were normalised to 1.0 at the point of addition of DOC. Left panel shows pCsgG *vs.* pCsgG/pCsgC co-expression. Right panel shows pCsgEFG *vs.* pCsgEFG/pCsgC co-expression. To confirm expression of CsgC and CsgG, samples from the t=140 minute time point were analysed by Western blotting using anti-6xHis antibodies (shown inset).

Supplemental Experimental Procedures

$CsgG_{16\text{-}277}$ sample preparation. Recombinant protein was expressed as described for full length CsgG-6xHis (see main methods section). Cells were harvested by centrifugation, lysed by the cell disruptor and the soluble fraction obtained through centrifugation at 18,000 rpm for 25 min at 4 °C. The clarified supernatant was incubated with Ni-NTA resin for 30 min before being separated from the resin. The resin was washed extensively with a buffer containing 20 mM Tris-HCl, 300 mM NaCl, 50 mM imidazole until the A280 nm value reached zero. $CsgG_{16\text{-}277}$ was then eluted from the column with 10 column volumes of Tris/NaCl buffer containing 300 mM imidazole.

NMR spectroscopy. $^{13}C^{15}N$-labelled CsgC was expressed in standard M9 minimal medium and purified as described in the main methods section. Data was collected using a Bruker Avance 600 MHz spectrometer, processed by NMRPipe (Delaglio *et al.*, 1995) and analysed in NMRDraw. Assignment of backbone nuclei was achieved by analysis of HNCA, HNCACB, HNCO, and CBCACONH spectra using a combination of manual and automated assignment by Mars (Jung and Zweckstetter, 2004). SO-FAST HMQC (Schanda *et al.*, 2005) spectra were used to compare chemical shift changes between the oxidised and reduced states of CsgC. Backbone amide relaxation rates were obtained through R1, R2 and heteronuclear NOE measurements.

Thermal shift assay. The thermal melting (T_m) point of CsgC was monitored using a ThermoFluor style assay (Niesen *et al.*, 2007). Five solutions of 20 µl were set up containing 30 µM CsgC (oxidised or reduced) in 10 mM HEPES pH 7.2, 0.1 M NaCl, 1 mM EDTA, 1X Sypro Orange dye. Fluorescence was recorded whilst heating the samples at a rate of ~1 °C per minute from 15 to 99 °C. T_m values were calculated by performing non-linear least-squares fitting to the data as described previously (Niesen *et al.*, 2007).

Circular dichroism. CD spectra of CsgG-6xHis at 0.1 mg/ml in 10 mM sodium phosphate pH 7.8, 100 mM sodium fluoride, 0.54 mM DDM were acquired using a Chirascan spectrometer (Applied Photophysics, 1 mm quartz cuvette). Triplicate scans were recorded across the range 260-182 nm, averaged, corrected by subtraction of buffer spectra, and smoothed using a 3-point moving average within Excel (Microsoft).

Size-exclusion chromatography of detergent-solubilised CsgG-6xHis. Approximately 2 mgs of CsgG-6xHis was loaded onto a Superdex 200 16/60 gel filtration column equilibrated in 20 mM Tris-HCl pH 8.0, 150 mM NaCl, 0.54 mM DDM. The protein was eluted at a rate of 0.5 ml/min and peaks positions were compared to the elution profiles of known standards.

Size-exclusion chromatography of $CsgG_{16\text{-}277}$. ~1 mg of $CsgG_{16\text{-}277}$ was loaded onto a Superdex 200 10/300 gel filtration column equilibrated in 20 mM Tris-HCl pH 8.0, 150 mM NaCl. The protein was eluted at a rate of 0.5 ml/min and peaks positions were compared to the elution profiles of known standards (Thryoglobulin, 669 kDa; Ferritin, 440 kDa; β-amylase, 200 kDa; Alcohol dehydrogenase, 150 kDa; bovine serum albumin, 66 kDa; carbonic anhydrase, 29 kDa, GE Healthcare).

Native gel electrophoresis of CsgG-6xHis. A ten microlitre sample containing ~10 µg CsgG-6xHis in 20 mM Tris-HCl pH 8.0, 150 mM NaCl, 0.54 mM DDM was mixed with 2 µl 5% (w/v) Coomassie G-250 sample additive and 4 µl 4X NativePAGE Sample buffer (Invitrogen) loaded onto a 4-16% Bis Tris polyacrylamide gel (Invitrogen), electrophoresed and stained according to the manufactuer's instructions relevant to the analysis of membrane proteins. High molecular weight markers (#17-0615-01, GE Healthcare) were run to assist in the sizing of the CsgG oligomer.

Bile salt sensitivity of *E. coli* cells overexpressing Csg proteins. C41 (DE3) cells harbouring a pET28 plasmid expressing CsgG-6xHis or CsgEFG-6xHis with or without a pBADMycHisC plasmid carrying the *csgC-6xHis* gene were cultured overnight in Terrific Broth (TB). The cultures were normalised by OD_{600} and used to inoculate 5 ml fresh TB and grown at 37 °C until mid-log phase (OD_{600nm} ~0.6 units). Cultures were then cooled to 26 °C and recombinant protein expression was induced by 0.2 mM IPTG and 0.01% (w/v) arabinose as appropriate. After 45 minutes sodium deoxycholate (DOC) was added to a final concentration of 0.1% (w/v). Culture densities were monitored by taking absorbance measurements at various time points, using appropriate dilutions to

ensure data accuracy at high cell densities. To confirm recombinant protein expression a 0.25 ml aliquot of cells was taken 140 minutes post-expression, lysed by B-PER II (Pierce), and subjected to SDS-PAGE and Western blotting using anti-6xHis antibodies (Sigma).

Supplementary References

Delaglio, F., Grzesiek, S., Vuister, G.W., Zhu, G., Pfeifer, J., and Bax, A. (1995). NMRPipe: a multidimensional spectral processing system based on UNIX pipes. J Biomol NMR *6*, 277-293.

Jung, Y.S., and Zweckstetter, M. (2004). Mars -- robust automatic backbone assignment of proteins. J Biomol NMR *30*, 11-23.

Niesen, F.H., Berglund, H., and Vedadi, M. (2007). The use of differential scanning fluorimetry to detect ligand interactions that promote protein stability. Nat Protoc *2*, 2212-2221.

Schanda, P., Kupce, E., and Brutscher, B. (2005). SOFAST-HMQC experiments for recording two-dimensional heteronuclear correlation spectra of proteins within a few seconds. J Biomol NMR *33*, 199-211.
